# Is mood associated with perception of recovery? Preoperative depression versus postoperative delirium after cardiac surgery

**DOI:** 10.1093/icvts/ivac229

**Published:** 2022-09-01

**Authors:** Ari A Mennander

**Affiliations:** Tampere University Heart Hospital, Tampere University, Tampere, Finland

**Keywords:** Cardiac surgery, Depression, Delirium

## Abstract

The author alone is responsible for the Invited Commentary, which does not necessarily reflect the policy of the Journal.

It is tempting to assess outcome after cardiac surgery by evaluating mortality, need for reoperations and dialysis, strokes and complications. Many important outcome variables are usually considered dichotomous either-or not- variables and easy to quantify, but from the patient’s point of view, success after surgery is also determined by neuropsychologic recovery [1]. Anxiety, depression and delirium are also major criteria that affect immediate recovery and require management, especially after major surgery [1, 2].

Excessive alcohol consumption, drug abuse and the presence of comorbidities are traditionally considered risk factors of depression and delirium [3]. While identifying depression in a patient is yet a clinical challenge despite some objective diagnostic criteria, perceiving depressive symptoms is also dependent on individual experience [1]. Major trauma and sensitivity to disease may elicit both depression and delirium [2]. Depression may be more frequent among patients diagnosed with cardiovascular disease than in the general population [3]. The risk of delirium is also considered enhanced by age [4].

Falk *et al.* [5] hypothesized that the presence of depression is associated with delirium after cardiac surgery. Based on a population-based cohort study with 1133 patients undergoing cardiac surgery patients in Stockholm 2013–2016, the authors investigated the association between preoperative depression and postoperative delirium using multivariable logistic regression analysis adjusted for variables clinically relevant for delirium. The study encompassed a patient questionnaire prior to surgery, the patient baseline characteristics were included in a data register, and postoperative delirium was evaluated by assessing medical records for symptoms of postoperative delirium according to the Diagnostic and Statistical Manual of Mental Disorders (DSM-5) criteria. The study was performed using the Strengthening the Reporting of Observational studies in Epidemiology (STROBE) checklist for cohort studies.

Preoperative depression was present in 14%, and postoperative delirium occurred in 26% of the patients, especially among the elderly patients. Postoperative delirium was diagnosed in 34% of the patients with preoperative depression as opposed to only 24% of the patients without depression. The incidence of delirium was more than double in patients with versus without preoperative depression according to adjusted multivariable analysis. The authors suggested that screening of preoperative depression may enhance awareness of early management of postoperative delirium.

The current study by Falk *et al.* [5] is well-written and comprehensive. The patients were identified using a unique personal identity number available for the citizens in Sweden. The limitations of the study were clearly discussed. Delirium was identified using a Confusion Assessment Method and patient registry files. Patients were grouped and compared within different ages. Early extubation after surgery allowed identification of early delirium. Patient care was exemplary and included cardiopulmonary bypass under standard anaesthesiologic care encompassing routine preoperative and postoperative surveillance.

It was beyond the scope of the retrospective design of the study to investigate causality of depression versus delirium. The diagnosis of delirium was based on early symptoms without specific imaging techniques to rule out possible strokes or embolisms. The effect of treatment, the onset and duration of depression, or delirium were not studied. Delirium was recorded after surgery, but without follow-up after hospitalization. More than half of the patients did not fill-in the questionnaire before surgery, but only 13 patients (1.2%) were excluded due to mortality, cancelling of surgery or postoperative unconsciousness.

Interestingly, the mean age for preoperatively depressed patients was only 62 vs 66 years in the non-depressed patients. The depressed patients may have had lower control of life than the non-depressed patients. According to the study, depressed patients under 62 years of age had significantly higher risk of developing delirium as compared with the rest of the patients. The study shows that a depressed 60-year-old patient had the same risk of developing delirium after cardiac surgery as a non-depressed 75-year-old patient. May be a competing risk factor analysis could further elaborate whether some patients may die or experience stroke during follow-up; delirium may impact recovery after cardiac surgery including cognitive decline, quality of life, hospital readmissions, increased mortality and increased costs.

Risk stratification of the patient undergoing cardiac surgery includes preoperative analysis of patient characteristics. Excellent surgical technique alone suffices not for satisfying outcome, one also expects supportive patient care during hospitalization. The current study by Falk *et al.* [5] importantly confirms that depression may hint for poor outcome after cardiac surgery [6]. The challenge is to quantify and define depression before and after surgery to further investigate follow-up outcome after surgery [1, 6]. We may recognize the patient with depression and delirium in practice, but traditionally the gravity of these entities has not been specifically quantified during or after hospitalization [1]. A holistic and patient-centred approach may need to be considered also during cardiac surgery. Depression may not only be a dichotomous either-or not- variable but may also be considered incrementally associated with outcome after cardiac surgery.

**Conflict of interest:** none declared.

## References

[1] Mollon B , Mahure SA, Ding DY, Zuckerman JD, Kwon YW. The influence of a history of clinical depression on peri-operative outcomes in elective total shoulder arthroplasty: a ten-year national analysis. Bone Joint J 2016;98-B:818–24. 27235526 10.1302/0301-620X.98B6.37208

[2] Radinovic KS , Markovic-Denic L, Dubljanin-Raspopovic E, Marinkovic J, Jovanovic LB, Bumbasirevic V. Effect of the overlap syndrome of depressive symptoms and delirium on outcomes in elderly adults with hip fracture: a prospective cohort study. J Am Geriatr Soc 2014;62:1640–8. 25243678 10.1111/jgs.12992

[3] Skajaa N , Adelborg K, Horváth-Puhó E, Rothman KJ, Henderson VW, Thygesen LC et al Stroke and risk of mental disorders compared with matched general population and myocardial infarction comparators. Stroke 2022;53:2287–98. 35317610 10.1161/STROKEAHA.121.037740

[4] Mahanna-Gabrielli E , Zhang K, Sieber FE, Lin HM, Liu X, Sewell M et al Frailty is associated with postoperative delirium but not with postoperative cognitive decline in older noncardiac surgery patients. Anesth Analg 2020;130:1516–23. 32384341 10.1213/ANE.0000000000004773 PMC7875454

[5] Falk AS , Kåhlin J, Nymark C, Hullgren R, Stenman M. Depression is associated with delirium after cardiac surgery—a population-based cohort study. Interact Cardiovasc Thorac Surg 2022;35(2):ivac151. 10.1093/icvts/ivac151 PMC9297521 35640560

[6] Tully PJ , Baker RA, Winefield HR, Turnbull DA. Depression, anxiety disorders and type D personality as risk factors for delirium after cardiac surgery. Aust N Z J Psychiatry 2010;44:1005–11. 21034183 10.3109/00048674.2010.495053

